# Desmoid Tumours of the extremity and trunk. A retrospective study of 44 patients

**DOI:** 10.1186/s12891-017-1924-3

**Published:** 2018-01-05

**Authors:** Laura Wirth, Alexander Klein, Andrea Baur-Melnyk, Thomas Knösel, Lars H. Lindner, Falk Roeder, Volkmar Jansson, Hans Roland Dürr

**Affiliations:** 1 Musculoskeletal Oncology, Department of Orthopaedic Surgery, Physical Medicine and Rehabilitation, Campus Grosshadern, University Hospital, LMU Munich, Marchioninistr. 15, 81377 Munich, Germany; 2 Institute of Radiology, Campus Grosshadern, University Hospital, LMU Munich, Marchioninistr. 15, 81377 Munich, Germany; 3 Institute of Institute of Pathology, Campus Grosshadern, University Hospital, LMU Munich, Marchioninistr. 15, 81377 Munich, Germany; 4 Department of Medicine III, Campus Grosshadern, University Hospital, LMU Munich, Marchioninistr. 15, 81377 Munich, Germany; 5 Department of Radiation Oncology, Campus Grosshadern, University Hospital, LMU Munich, Marchioninistr. 15, 81377 Munich, Germany; 6 CCU Radiation Oncology, German Cancer Research Center (DKFZ), Heidelberg, All authors are part of the SarKUM, the Bone and Soft Tissue Tumor Center of the University Hospital, Ludwig-Maximilians-University Munich, Munich, Germany

**Keywords:** Fibromatosis, Extra-abdominal, Desmoid, Recurrence, Prognosis

## Abstract

**Background:**

Desmoid-type fibromatosis (DF) is a aggressive (myo)fibroblastic neoplasm with an infiltrative growth and a tendency to local recurrence. Resection of the tumour and/or radiation were proposed as principal treatment. The aim of this retrospective study was to analyze the local control rates focusing on the effect of surgical margins and radiotherapy.

**Methods:**

From 1981 to 2014, 44 patients had been treated. Fifty four therapies had been applied, in 50 cases surgery +/− radiation therapy, NSAIDs or chemotherapy. In 4 cases a conservative approach was chosen. Thirty seven patients had primary, 17 recurrent disease. Endpoint was either local recurrence (LR), progression of residual disease or rare non-metastatic secondary lesions at the same extremity.

**Results:**

The mean age was 39,4 years. In 17 cases a R0, in 27 a R1 and in 6 cases a R2 resection was achieved. Four patients were treated conservatively. All together in 21 cases radiotherapy, in 5 NSAIDs, in 3 imatinib and in 2 cases each tamoxifen or chemotherapy had been applied. The median follow-up was 119 months. 5-year recurrence free survival after resection was 78%. 10 (20.4%) patients developed LR between 5 and 42 months after therapy. Recurrent disease was a negative factor on LR. Margins, radiotherapy, sex, or size of the tumour had no significant impact on LR. Patients younger than 40 years had a significant higher risk of LR.

**Conclusions:**

Surgical margins are less important than keeping function. Radiotherapy might be an option in unresectable lesions, the role of adjuvant radiotherapy is controversially discussed.

## Background

Desmoid-type fibromatosis (DF) is defined by the World Health Organization (WHO) as a locally aggressive (myo)fibroblastic neoplasm arising usually in deep soft tissues with an infiltrative growth and a tendency to local recurrence, but a lack of metastatic potential. [1] The incidence is estimated 2.1–5.4 per million people per year increasing in both locations, extra-abdominal and in the abdominal wall. [2] There are two group of patients with prognosis quite different between both. Patients there DF is associated with the autosomal dominant familial adenomatous polyposis (FAP) syndrome characterized by a germline mutation of the adenomatous polyposis coli (APC) gene and a risk of 30% developing DF and patients with sporadic DF (harboring the CTNNB1 mutation (beta-catenin) in the tumor, which are considered mutually exclusive. [3]

DF are most often seen between 16 and 60 years of age twice as common in female than male patients. [4] Extra abdominal Desmoid fibromas can affect any anatomical region but are most common in the limb or the limb girdle (50%), the trunk (43%) or the head and neck region (7%). [5] 10% of cases had been described as multifocal. [6]

In former years resection of the tumour and/or radiation were proposed as principal treatment. [7] Systemic medical treatment including non-steroidal anti-inflammatory drugs (NSAIDs), anti-estrogens, cytotoxic chemotherapy, Interferon α or tyrosine kinase inhibitors showed conflicting results. [8] With active surveillance alone a spontaneous regression of 28–50% of cases in extra abdominal DF is observed thus interfering with outcome data of any treatment studies. [4] Therefore the European Sarcoma Network Working Group (ESNWG), the European Organization for Research and Treatment of Cancer (EORTC) and others consider an initial period of active observation in extra abdominal DF as proposed already more than a decade ago by some authors. [8–11]

The aim of this retrospective study was to analyze the local control rates in a consecutive single-institution series of surgically-treated patients focusing on the effect of surgical margins and adjuvant radiotherapy in primary and recurrent disease.

## Methods

From 1981 to 2014, 44 consecutive patients with DF of the extremities and trunk wall had been treated in our institution. In those 44 patients in total 54 therapies had been done, in 50 cases surgery +/− additional therapies as radiation therapy, NSAIDs or chemotherapy. In 4 cases a conservative approach was chosen. All tumours had a diagnosis of DF based on histological features and immunohistochemistry. Thirty seven patients had primary, 17 recurrent disease. Five of the recurrences developed in 5 patients with primary resections included also in this study. Seven patients had recurrences after first treatment elsewhere. From those 7 patients 3 there treated a second time for a further recurrence and one for a second and third recurrence.

Preoperative staging included MRI (predominantly) or CT of the tumour region. Tumour size was determined by the greatest diameter of the tumour in preoperative imaging. All patients underwent limb-sparing surgical resection. The margin was defined as R0 if a rim of sound tissue around the lesion was present (wide resection), R1 if the margins were contaminated by the tumour (marginal resection) or R2 if remaining tumour was evident (intralesional resection). In one case follow-up could not be obtained. Fifty four cases were followed routinely for evidence of local recurrence or secondary lesions, also preferably with MRI.

For statistical analysis, overall and recurrence-free survival were calculated by the Kaplan-Meier method. Univariate subgroup analysis was done using the log-rank test (time-to-event data) or the chi-square test. For multivariate analysis, a Cox proportional-hazard regression model was used. Significance analysis was performed using the log-rank test, the Chi-Square test, or the Cox proportional-hazards regression. The data analysis software used was MedCalc®.

## Results

The patients details are described in Table 1. In 4 cases, multiple lesions were evident. None of our patients had FAP-associated disease but routine GI screening (colonoscopy) was not done.

**Table 1:** Patient characteristics

Number of patients	44
Sex	25 f, 19 m
Mean age (range)	39.4 y (14–69)
Number of treatments	54
Mean tumour size (range)	7.7 cm (1–25)
Tumour site n(%)	
Upper limb	21 (39%)
shoulder	9
upper arm	7
lower arm	2
axilla	2
hand	1
Lower limb	19 (35%)
upper calf	7
lower calf	5
foot	6
knee	1
Trunk	8 (15%)
Pelvis	6 (11%)
Presentation	
Primary	37 (69)
Recurrent	17 (31

The mean duration of symptoms in primary disease was 13.2 months (range, 1–96), in recurrent cases 4.6 months (range, 1–12) n.s. 31 (62%) patients complained of swelling, 29 (58%) of pain. Neurological impairment (sensory) or restriction of movement was seen occasionally.

In 17 cases a wide (R0) resection, in 27 a marginal (R1) resection and in 6 cases an intralesional (R2) resection was achieved. Only one patient had a minor amputation (distal toe). In 4 patients (all recurrent tumours) no resection but either radiotherapy, NSAIDs, imatinib or NSAIDs/tamoxifen had been applied. All together in 21 cases radiation therapy, in 5 cases NSAIDs, in 3 cases imatinib and in two cases each tamoxifen or cytotoxic chemotherapy had been applied.

**Fig. 1:**
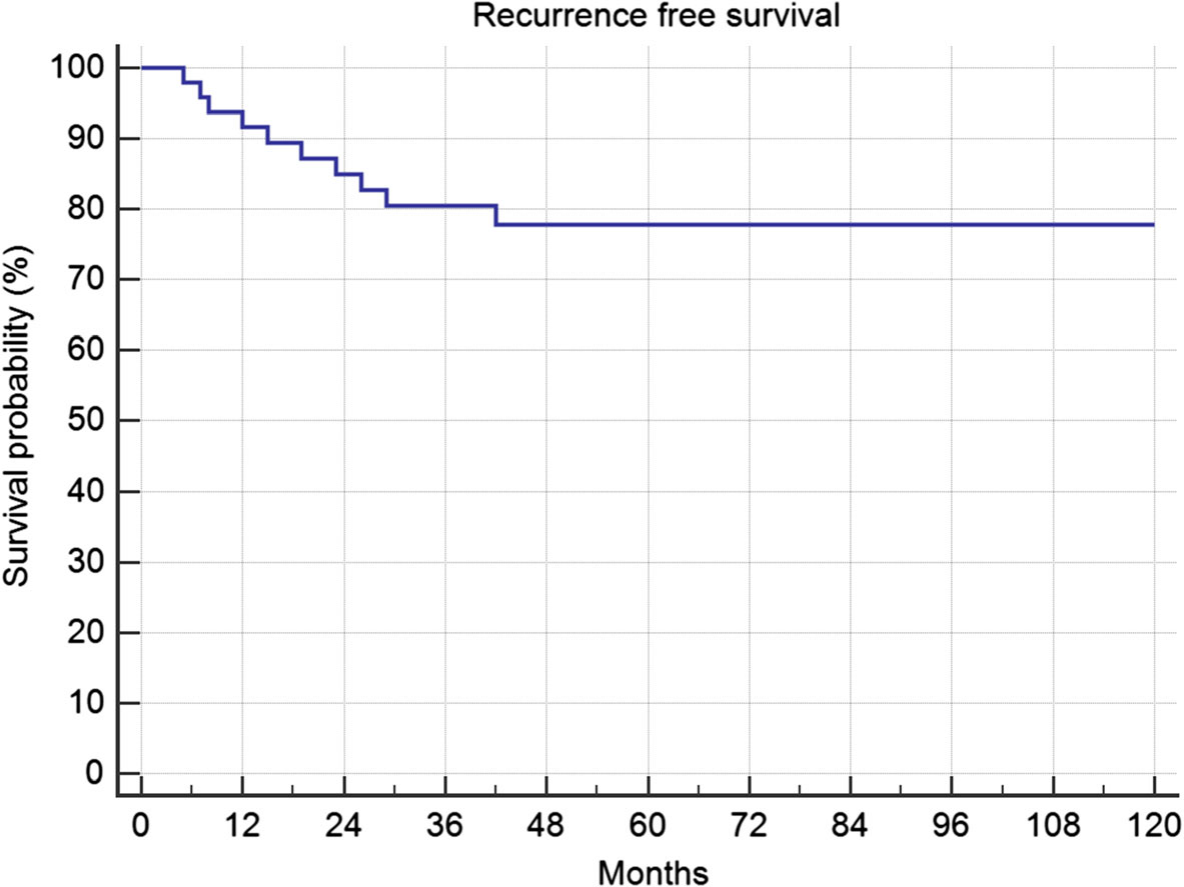
Recurrence free survival after 49 resections for DF

One patient with surgical resection was lost to follow-up, leaving 49 resected and 4 conservative treated cases. Two patient died in follow-up independent to the tumour or therapy. The median follow-up was 119 months (range, 3–412). Only 2 patients had a follow-up of less than 12 months. Over all 5-year recurrence free survival after resection was 78% (Fig. 1). In total 10 (20.4%) patients developed local recurrences between 5 and 42 months after therapy.

**Fig. 2:**
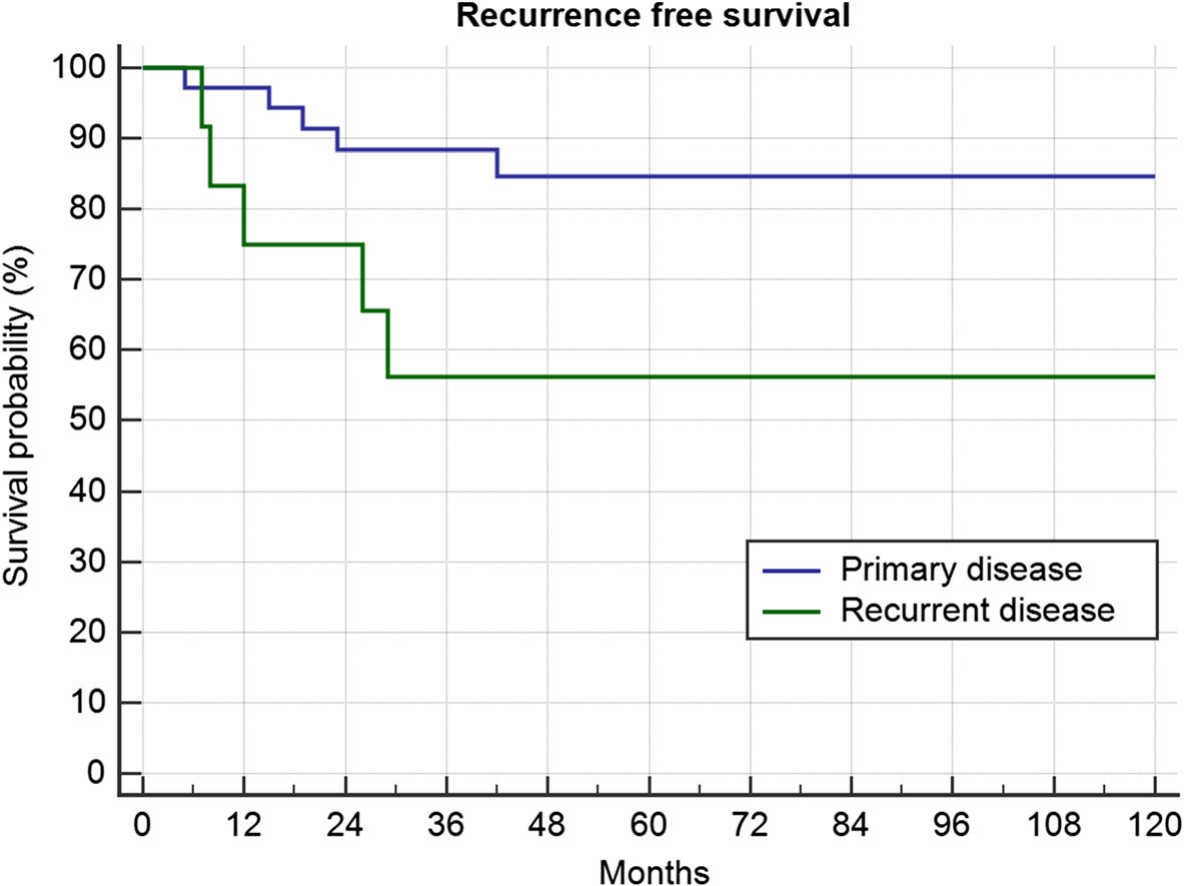
Recurrence free survival in 37 primary and 12 recurrent resections (*p* = 0.0338)

In every case the indication of adjuvant radiotherapy was discussed in dependence to the achieved margins and the approximity of functionally important structures. As shown in Table 2 recurrent disease was a negative factor on LR (Fig. 2; *p* = 0.0338). Of the 6 patients with intralesional surgery, one was lost to follow-up. One patient has a stable disease after radiation, another 2 patients also a stable disease without adjuvant therapy. One patient is progressive, starting 2 years after surgery, without further treatment, one patient is treated with imatinib and chemotherapy for now 3 months.

**Table 2:** Summary of local recurrence in surgically treated patients

Total	Primary		Recurrent		R0		R1		R2	
	-RTX	+ RTX	- RTX	+ RTX	-RTX	+ RTX	-RTX	+ RTX	-RTX	+ RTX
10/49 (20.4%)	4/24	1/13	1/6	4/6	2/12 (16.7%)	1/5 (20%)	3/14 (21.4%)	4/13 (30.8%)	0/4 (1 Progression)	0/1
	5/37(13.5%)		5/12 (41.7%)							
	*p* = 0.0374				n.s.		n.s.		n.s.	

*RTX* Radiotherapy, *R0, R1, R2* Resection margin

In univariate analysis margins, radiotherapy, sex, or size of the tumour did not show a significant impact on local recurrence.

**Fig. 3:**
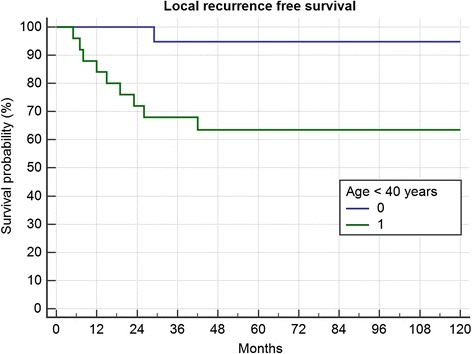
Recurrence free survival in 26 resections in patients <40 years and 23 resections in patients >40 years (*p* = 0.0104)

From 49 evaluable surgically treated cases 26 were younger than 40 years. Those patients had a significant higher risk of local recurrence (Fig. 3; *p* = 0.0104). Only one patient in the group of patients older than 40 years showed recurrent disease. In multivariate analysis (primary/recurrent, age < 40 years) only age kept significance. (PD/RD *p* = 0.11, HR 2.80, CI 0.8–9.8; Age *p* = 0.0495, HR 8.0, CI 1.0–63).

From the 4 patients with conservative therapy only, all had had recurrent disease. One is progressive after radiotherapy, one shows stable disease for more than 8 years after initial NSAIDs, another one after imatinib and the last patient has no evidence of disease after NSAIDs and tamoxifen.

## Discussion

Diagnosis and imaging in DF is well described. [4, 8] Most of the cases of DF are sporadic. Metachronous or synchronous multicentric disease is seen in up to 10% of cases. [7] In the last years a number of clinical factors as e.g. age, size and site had been identified for progression free survival. [12] Despite being considered “benign”, DF bears a considerable risk of local recurrence and functional impairment. Surgical resection, which had been the mainstay of therapy over a long period of time is associated with local recurrence rates as high as 19–75%, but in most of the studies below 50%. [5, 7, 13–19] Regarding the margins which should be obtained there are contradictory recommendations mainly based on institutional experience. There are studies which prove a lower rate of recurrent disease in R0 resected cases. [13, 19] In a major review of the literature in 2004 by Leithner et a. the authors included 12 studies with 412 primary and 127 recurrent cases. [20] In primary cases wide or radical resections showed a recurrence rate of 27%, in marginal or intralesional cases 72% developed local recurrences (*p* < 0.001). In recurrent cases the numbers were 49 and 88% respectively (*p* < 0.001). Four years later a second metaanalysis with the same focus was published. [21] From 17 studies a significant effect of margin on recurrence was seen in 7, a trend in 5, and in 5 studies marginal status did not affect the local recurrence rate as e.g. 23% for R0 vs. 26% for R1 in primary resections. [22] So our own results of 18% for R0 and 26% for R1 resections fit well into this scheme as also published in more recent studies. [18] Most impressive was the high frequency of further recurrences in recurrent disease in this study (42%). As shown above this is a well known fact as described in the metaanalysis by Leithner et al. Even in wide or radical resections in those patients 49% recurred. There are a number of studies coming to the same conclusion [15, 17] but there are also conflicting results. [13, 23] Identical recurrence rates of 33% are reported in 85 primary and 104 recurrent cases [24] whereas others even found a positive trend of local recurrence with 75% after primary 53% after first and 50% after second recurrence. [25] But as for the majority of the literature a surgical approach in recurrent disease should be avoided. In addition in some of the reviewed studies margins at all lost significance in multivariate analysis. The authors conclude, that functionally disabling operations to obtain negative margins should in any cases be avoided.

Younger age seems to deteriorate the results as also shown in this study. [13, 18] Shin et al. described in 60 patients younger than 38 years local recurrence as 40% vs 16% in patients older than 38 years. [15] Patients age older than 37 years proved to be statistically associated with longer progression free survival as shown by Salas et al. [12] Interestingly the authors state that the identification of biologic pathways involved in the tumorigenesis of desmoids emphasized these age differences, genomic alterations being more common in older patients. [26]

Radiotherapy has a major role in the treatment of soft tissue sarcomas. But as surgery, this is not as clear in DF. A major problem in most of the retrospective studies, as in ours, is a mixing of patients with different presumed risk factors in irradiated and not irradiated treatment groups. Especially margin status, as described above an itself debatable prognostic factor, is interfering strongly with radiation data. Striking in our study was the high recurrence rate of 4/6 patients irradiated after recurrent disease as compared to 1/13 in primary disease. The role of radiotherapy in DF management remains controversial and is extensively debated. [27]

Ballo et al. showed a 10-year recurrence rate of 23% with combination of surgery and radiotherapy and 31% with radiotherapy alone (n.s.). [28] A dose of 50 Gy was associated with a 60% relapse rate, whereas higher doses yielded a 23% relapse rate (*p* < 0.05). This effect was also seen in a larger retrospective multicenter review. [29] In a second publication Ballo et al. showed a 10-year recurrence rate of 54% with surgery alone in R1 resected patients, 27% in R0 resected patients, 25% in combination of surgery and radiotherapy and 24% with radiotherapy alone. [24] Spear et al. reported an overall recurrence rate of 31% in surgery treated patients alone vs. 28% combined with radiation (n.s.). [30] In 23 R0 resections they showed a trend for lesser recurrence with additional radiation (22% vs. 44%) but without significance. Other authors failed, as in our series, to demonstrate any significant benefit of adjuvant radiotherapy, even accounting for margin status. [5, 13, 21–23, 31] In a review of 22 studies by Nuyttens et al., in 2000, in 234 patients treated with surgery alone and 80 patients treated with radiotherapy alone, local recurrence was 39% vs 22% (*p* = 0.023). [32] As described by Shin et al. or Houdek et al. adjuvant radiotherapy delayed the recurrence of the tumour without having an effect on the ultimate relapse rate. [15, 33] In a retrospective multicentric European review of 110 patients local recurrence in surgery only was 32% vs. 25% after combined treatment. Local progression free survival proved to be better in combination (*p* < 0.001). [29] In a prospective EORTC and ROG study published in 2013 44 patients received 56 Gy. [34] 10 patients (23%) developed local progression, 5 patients developed new lesions. Based on the specific growth pattern, field definition is critical. In 6 recurring patients after radiation therapy only one patient had a true in-field recurrence. [35] In another 17 patients, 7 recurrences were seen at the field border. [29] The impact of radiotherapy in the treatment of desmoid tumours remains hence unclear. Considering adjuvant radiotherapy, the data is even less clear. Functional complications after radiotherapy are well known, due to the in general unimpaired life expectancy in DF also radiation-induced malignancies should be considered. [36]

In the last years, a watchful “Wait-and-See” policy has been advocated by many authors. In a retrospective survey on 55 patients Briand et al. described a 85% spontaneous growth arrest. [37] Half of the tumours were stabilized at 1 year, one case increased continuously beyond 3 years, 2 patients showed a regrowth. In 20 patients treated with surveillance only, after median 35 months one complete and 5 partial regressions and 13 stable diseases were seen. Only one patient progressed needing surgery. [38] In a retrospective study of 109 patients initially treated by observation, 51 required interventions as they progressed. [39]

So a more conservative approach seems to be justified and is at the moment part of prospective trials or current treatment guidance. [4, 8]

## Conclusion

In summary DF has a high rate of recurrence. As published in the last years spontaneous growth arrest or even regression of the tumour are in numbers comparable to the effect of surgical resections. If surgery is necessary, surgical margins are less important than keeping function for the patient. There was no difference between R0 and R1 resections in Local recurrence free survival. Other treatment modalities should be preferred over surgery when DF ultimately recurs.

Definitive radiation might be an option in unresectable progressive lesions, the role of adjuvant radiotherapy is controversially discussed. Younger age and recurrent disease seem to increase the risk of (further) local recurrence. A watchful “Wait-and-see” policy in primary lesions seems to be justified by the published data.

## Abbreviations

APC: Adenomatous polyposis coli
CI: Confidence interval
Cm: Centimeter
CT: Computed Tomography
CTNNB1: Catenin (Cadherin-Associated Protein), Beta 1 gene
DF: Desmoid-type fibromatosis
EORTC: European Organization for Research and Treatment of Cancer
ESNWG: European Sarcoma Network Working Group
f: Female
FAP: Familial adenomatous polyposis
GI: Gastrointenstinal
HR: Hazard ratio
LR: Local recurrence
M: Male
MRI: Magnetic resonance imaging
NSAIDs: Nonsteroidal antiinflammatory drugs
R0, R1, R2: Resection margin
RTX: Radiotherapy
WHO: World Health Organization
Y: Years
